# Femoral derotational osteotomies for version abnormalities: a mean ten-year follow-up

**DOI:** 10.1302/2633-1462.75.BJO-2026-0014.R1

**Published:** 2026-05-20

**Authors:** Brian T. Muffly, Zachary A. Trotzky, Branden R. Sosa, Olivia M. Jochl, S. Robert Rozbruch, Robert L. Buly

**Affiliations:** 1 Hospital for Special Surgery, New York, New York, USA; 2 Department of Orthopaedic Surgery, Emory University, Atlanta, Georgia, USA

**Keywords:** Femoral anteversion, Femoral retroversion, Femoral derotational osteotomy, Hip preservation, Femoral version, osteotomies, hips, arthroplasty, excessive femoral anteversion, deformities, patient-reported outcome measures (PROMs), hip arthroscopy, periacetabular osteotomy, modified Harris Hip Score, intramedullary device

## Abstract

**Aims:**

This study aimed to characterize long-term patient-reported outcomes and survivorship following femoral derotational osteotomy (FDO).

**Methods:**

A total of 77 hips (60 patients) between March 1997 and February 2019 who underwent FDO using an antegrade intramedullary device for excessive femoral anteversion or retroversion by a single-surgeon were retrospectively reviewed. Those with minimum five-year follow-up, with a mean of ten years (5.2 to 26.8), were included. Pre/postoperative modified Harris Hip Score (mHHS), postoperative patient global impression of change (PGIC), and patient acceptable symptomatic state (PASS) responses were collected. Survivorship free of conversion to arthroplasty was determined.

**Results:**

Overall, 90.0% of patients were female with a mean age of 29.6 years (14.4 to 60.9). A total of 52 hips had excessive anteversion with mean deformity of 38.3° (22° to 58°) and mean correction of 22.4° (15° to 40°); 25 hips had excessive retroversion with mean deformity of -8.7° (-24° to 2°) and mean correction of 21.7° (15° to 35°). Concomitant procedures included hip arthroscopy (32.5% of cases), periacetabular osteotomy (9.0%), and tibial derotational osteotomy (7.8%). Regardless of deformity, mean mHHS significantly improved (61.6 to 89.7, p < 0.001). The proportion of minimal clinically important difference (MCID), substantial clinical benefit (SCB), and PASS achievement was 94% (72 of 77), 84% (65 of 77), and 87% (67 of 77), respectively. For PGIC, 95.2% reported improvement, including 88.7% who reported themselves ‘very much improved’ or ‘much improved’. Overall, 92.7% of anteverted and 85.7% of retroverted patients found their current symptomatic state satisfactory (p = 0.398). Three hips (two patients) underwent arthroplasty at mean 4.3 years. Survivorship free from conversion to arthroplasty was 95% at ten, 15, and 20 years.

**Conclusion:**

In appropriately selected patients, FDO for hip pain related to excessive femoral anteversion or retroversion provides significant improvements in pain and function at a mean ten-year follow-up. Most patients report postoperative improvement and find their current symptomatic state satisfactory. The procedure effectively preserves the native hip joint with 95% long-term survivorship free of conversion to arthroplasty.

Cite this article: *Bone Jt Open* 2026;7(5):674–681.

## Introduction

Failure to recognize or insufficient correction of underlying structural deformities is a frequent cause of hip preservation procedural failure.^1-3^ Furthermore, several anatomical factors are often present contributing to chondrolabral degeneration.^4^ Abnormal femoral version (excessive anteversion or retroversion) is increasingly recognized as an aetiology of pain, dysfunction, and subsequent native hip joint degeneration. Among symptomatic hips, the prevalence of abnormal femoral version was recently reported to be 52%, including 17% of severe abnormalities.^5^ Excessive femoral anteversion or retroversion may exist in isolation or concurrently with acetabular dysplasia and/or femoroacetabular impingement (FAI).^4,6-8^ As such, evaluation of the painful pre-arthritic hip should be individualized, including a comprehensive history, physical examination, and advanced imaging to establish an accurate diagnosis and develop an appropriate treatment strategy.

Variations in femoral version have significant implications for hip biomechanics. These deformities alter the anteroposterior position of joint reaction forces during stance phase of gait.^9^ Increasing femoral anteversion decreases the abductor lever arm, necessitating more abductor muscle force to generate the required hip abduction moment,^10^ potentially manifesting as fatigue associated with prolonged standing/ambulation.^11^ Instability, chondrolabral complex damage, and development of eventual osteoarthritis have been observed with excessive anteversion.^12-14^ Affected patients are predisposed to posterior extra-articular impingement, including potential ischiofemoral impingement.^15,16^ Retroversion, conversely, can cause chondrolabral damage secondary to impingement. Such contact may ultimately result in hip osteoarthritis.^14,17^ These patients experience increased mean peak joint pressures,^18^ and are also at increased risk of slipped capital femoral epiphysis,^19,20^ traumatic posterior hip dislocations,^21^ as well as FAI arthroscopic treatment failure.^3^

The mainstay in treatment of excessive, symptomatic femoral anteversion or retroversion is the derotational osteotomy. While short-term results have been promising,^22-27^ longer-term results distinct from the neuromuscular population are relatively sparse. The purpose of this study was to characterize long-term patient-reported outcome measures (PROMs) and survivorship free of conversion to arthroplasty following femoral derotational osteotomy (FDO) for version abnormalities using an intramedullary device at a mean ten-year follow-up (5.2 to 26.8). The authors hypothesized that improvements in patient-reported pain and function, as well as high survivorship, would be observed.

## Methods

Following institutional review board approval, patients undergoing primary FDO between March 1997 and February 2019 for excessive femoral anteversion or retroversion by a single-surgeon at a tertiary academic medical centre with a mature hip preservation practice were retrospectively reviewed. Patients were indicated for surgery when all of the following criteria were met: functionally limiting hip pain failing conservative treatment measures in the setting of CT consistent with excessive femoral anteversion or retroversion (normal femoral version was considered 5° to 20°,^3^ with excessive considered outside of this range), radiographic joint space preservation (Tönnis grades 0 or 1),^28^ and clinical range of motion (ROM) (routinely measured in the supine position with the hip in 90° of hip flexion, and in the prone position at neutral hip flexion/extension) aberrations correlating with the specific version abnormality (e.g. excessive anteversion exhibiting significant internal rotation with minimal external rotation). Foot-progression angle alterations were common (e.g. excess anteversion demonstrating in-toeing and/or patellar in-pointing). MRI for chondrolabral evaluation, and CT scan were routinely obtained preoperatively. Using the Reikeras method, board-certified musculoskeletal radiologists performed femoral version measurements.^29,30^ Tibial torsion measurements were performed using axial images, with the proximal reference as the most prominent part of the posterior femoral condyle.^31,32^ The centre of the medial malleolus to centre of lateral malleolus was used as the distal reference.

Those without minimum five-year follow-up, or with presence of a coxa vara/valga deformity in which a derotational, intertrochanteric osteotomy was more appropriate were excluded. Overall, 77 hips (60 patients) met inclusion for analysis. Of these, 26 hips (33.8%) had failed a prior hip arthroscopy while one hip (1.3%) had undergone a prior combined hip arthroscopy and periacetabular osteotomy (PAO). Modified Harris Hip Score^33^ (mHHS; 100% response rate) was collected preoperatively and at latest follow-up, with higher scores (0 to 100 scale) indicating pain and functional improvement. Patient global impression of change (PGIC) and patient acceptable symptomatic state (PASS) responses (81.6% response rate for both) were assessed postoperatively.

### Patient characteristics

A total of 77 hips (60 patients) were analyzed: 90.0% were female with mean age of 29.6 years (SD 10.0; 14.4 to 60.9) (Table I ). The mean follow-up was ten years (5.2 to 26.8).

**Table I.** Demographic and clinical characteristics.

Variable	Study cohort (n = 77 hips, 60 patients)
Mean age, yrs (SD; range)	29.6 (10.0; 14.4 to 60.9)
Female, % (n)	90.0 (54)
Prior ipsilateral hip surgery, % (n)	48.1 (37)
Prior ipsilateral knee surgery, % (n)	7.8 (6)
**Aetiology, % (n)**	
Idiopathic	88.3 (53)
Post-traumatic	6.7 (4)
**Neuromuscular/syndromic, % (n)**	
Cerebral palsy	3.3 (2)
Prader-Willi syndrome	1.7 (1)

### Surgical technique

**Fig. 1:**
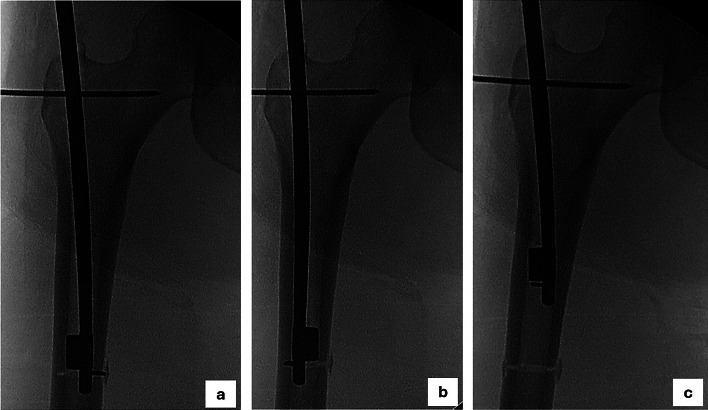
Intraoperative fluoroscopic images demonstrating osteotomy progression at the: a) medial and b) lateral (b) cortices, followed by c) osteotomy completion.

Femoral preparation for a trochanteric-entry intramedullary nail was performed. Utilizing triangles of known angular magnitudes, divergent Steinmann pins were placed in the desired amount of rotational correction. This value was predicated upon magnitude of deviation from normal version, clinical ROM, and foot-progression angle. The osteotomy was completed using an intramedullary hand saw (Intramedullary Bone Saw; Zimmer Biomet, USA). (Figure 1). The radius of the saw was progressively enlarged and rotated 360° under fluoroscopic guidance until osteotomy completion was observed. In the few cases where the intramedullary saw was unable to complete the osteotomy, typically in cases with small diameter femoral canal and thick lateral cortex, either a gentle valgus force was manually applied to the femur or percutaneous osteotome introduced to complete the osteotomy. Pins were then moved into parallel alignment through distal femoral segment rotation. Distal (dynamic) then proximal interlocking screws were placed, ensuring maintenance of rotational correction (maintained parallel alignment of aforementioned Steinmann pins) throughout. A more-normalized hip ROM profile was then confirmed.

Concurrent ipsilateral hip preservation procedures were performed in 49% (38/77) of cases (Table II). Concomitant hip arthroscopy was performed prior to FDO when imaging demonstrated large cam deformity and chondrolabral injury in the setting of planned, retroverting osteotomy that would move this lesion closer to mechanical conflict following derotation. Arthroscopy was also performed when an anteverting osteotomy would be insufficient to relieve mechanical conflict in the setting of a large cam lesion, or at patient request. Coexisting acetabular dysplasia was addressed with concurrent PAO. In the presence of compensatory external tibial torsion coexisting with excessive femoral anteversion,^14^ a concomitant tibial derotational osteotomy (TDO) was performed (just superior to the isthmus) to prevent an exaggerated external foot progression angle in patients lacking the in-toeing gait often associated with excessive femoral anteversion. Weightbearing was restricted to 20% for six weeks with concurrent PAO, otherwise patients were weightbearing as tolerated without ROM restrictions. Patients were routinely seen back at two weeks, six weeks, three months, and one year postoperatively, as well as annually thereafter.

**Table II.** Concomitant hip preservation procedures.

Procedure	Data	
Hip arthroscopy, % (n)	32.5 (25)	
Periacetabular osteotomy, % (n)	9.0 (7)	
Tibial derotational osteotomy, % (n)	7.8 (6)	
	**Preoperative**	**Postoperative**
Mean LCEA, ° (range)	17.5 (10 to 21)	32.3 (27 to 36)
Mean ACEA, ° (range)	20.6 (12 to 26)	38.5 (31 to 47)
External tibial torsion, ° (range)	44.8 (33.7 to 58.6)	

Mean lateral centre edge angle (LCEA) and anterior centre edge angle (ACEA) given for patients undergoing concurrent periacetabular osteotomy. External tibial torsion given for patients undergoing concurrent tibial derotational osteotomy.

### Statistical analysis

Shapiro-Wilk tests assessed normality. Mean mHHS improvement was calculated using Wilcoxon signed rank tests. Other continuous variables were analyzed by paired sample *t*-tests or Wilcoxon signed rank tests, as needed. Categorical variables were analyzed using chi-squared or Fisher’s exact test, as appropriate. One-way analysis of variance (ANOVA) assessed differences in PROMs by concomitant procedure. Thresholds for minimal clinically important difference (MCID), substantial clinical benefit (SCB), and PASS were determined using a receiver operating characteristic (ROC) curve and an area under the curve (AUC). MCID, SCB, and PASS were identified using the Youden index to maximize the sensitivity and specificity of the selected threshold on the ROC curve. Patients who responded ‘minimally improved’ or better on PGIC were considered to have achieved MCID. Patients who responded ‘much improved’ or better on PGIC were considered to have achieved SCB. Patients who responded ‘yes’ on the PASS question were considered to have achieved PASS. Survivorship was determined with Kaplan-Meier survival analysis. Significance was defined as a p-value < 0.05.

## Results

Aetiology of excessive femoral anteversion or retroversion was most commonly idiopathic (88.3% of cases). Excessive anteversion was present in 52 hips, with mean deformity of 38.3° (22° to 58°) and mean correction of 22.4° (15° to 40°; Table III). Clinically, this translated to a significant change in hip internal rotation from 60.8° preoperatively to 22.3° postoperatively, and external rotation from 28.6° to 46.7° (p < 0.001 for both). Femoral retroversion was present in 25 hips, with mean deformity of -8.7° (-24° to 2°) and mean correction of 21.7° (15° to 35°). This corresponded to significant changes in hip internal rotation from 7.6° preoperatively to 21.2° postoperatively, and external rotation from 79.2° to 42.2° (p = 0.006 and p= < 0.001, respectively).

**Table III.** Femoral version abnormalities.

Measure	Anteversion (n = 52 hips); mean (range)	Retroversion (n = 25 hips); mean (range)
Deformity, °	38.3 (22 to 58)	-8.7 (-24 to 2)
Correction, °	22.4 (15 to 40)	21.7 (15 to 35)
Postoperative version, °	15.9 (0 to 29)	13.0 (-2 to 22)
Preoperative internal rotation, °	60.8 (10 to 90)	7.6 (-20 to 30)
Postoperative internal rotation, °	22.3 (5 to 45)	21.2 (10 to 40)
Preoperative external rotation, °	28.6 (-5 to 60)	79.2 (50 to 100)
Postoperative external rotation, °	46.7 (15 to 60)	42.2 (30 to 50)
Preoperative AV 0100, °*	9.0 (-8.3 to 27.6)	-0.9 (-17 to 6.4)
Preoperative AV 0200, °*	15.8 (-3.7 to 34.7)	9.7 (-5.1 to 19.8)
Preoperative AV 0300, °*	20.4 (6.9 to 32.5)	14.9 (8.8 to 23.5)
Preoperative McKibbin Index, °*	60.2 (43.2 to 78.4)	3.8 (-11.7 to 14.4)

* Data present only for 34/77 hips (44.1%).

AV, acetabular version.

mHHS and PGIC/PASS response rates did not differ between anteversion and retroversion cohorts (100% vs 100%, p = 1.000; 78.8% vs 84.0%, p = 0.760, respectively; Table IV). Regardless of version abnormality, mHHS significantly improved pre- to postoperatively (61.6 vs 89.7, p < 0.001). mHHS outcome change thresholds for MCID and SCB were 3.6 (AUC 0.73) and 14.5 (AUC 0.87), respectively.

**Table IV.** Patient-reported outcome measures.

Outcome	Study cohort (n = 77 hips)	Anteversion (n = 52 hips)	Retroversion (n = 25 hips)
Preoperative mHHS, mean (SD)	61.6 (9.9)	60.6 (9.9)	63.5 (9.7)
Postoperative mHHS, mean (SD)	89.7 (12.8)	91.4 (11.0)	86.5 (15.6)
Improved (PGIC), % (n)	95.2 (59)	95.1 (39)	95.2 (20)
Very much improved (PGIC), % (n)	62.9 (39)	65.9 (27)	57.1 (12)
Much improved (PGIC), % (n)	25.8 (16)	24.3 (10)	28.6 (6)
Minimally improved (PGIC), % (n)	6.5 (4)	4.9 (2)	9.5 (2)
Satisfied (PASS), % (n)	90.3 (56)	92.7 (38)	85.7 (18)

mHHS, modified Harris Hip Score; PASS, patient acceptable symptomatic state; PGIC, patient global impression of change.

The mHHS postoperative score threshold for PASS was 74.8 (AUC 0.83). The proportion of MCID, SCB, and PASS achievement was 94% (72 of 77), 84% (65 of 77), and 87% (67 of 77), respectively. Overall, 95.2% reported improvement postoperatively, including 88.7% indicating themselves to be ‘very much improved’ or ‘much improved.’ PGIC improvement responses did not differ based on version abnormality. Overall, 90.3% of respondents find their current symptomatic state satisfactory, including 92.7% within the anteversion group and 85.7% in the retroversion group (p = 0.398).

No difference in mHHS change (p = 0.182) or proportion of positive PGIC/PASS responses (p = 0.516 and p = 0.673, respectively) was demonstrated when comparing isolated FDO with those undergoing concurrent preservation procedure. When stratified by specific concurrent preservation procedure, no differences were observed in the proportion of positive PGIC (p = 0.923) or PASS responses (p = 0.729). However, significant variation in mHHS change (p = 0.032) was seen between procedures: 25.5 (SD 14.5) for FDO in isolation, 26.5 (SD 13.0) for FDO with hip arthroscopy, 41.9 (SD 17.7) for FDO with TDO, and 37.1 (SD 18.5) for FDO with PAO.

**Fig. 2:**
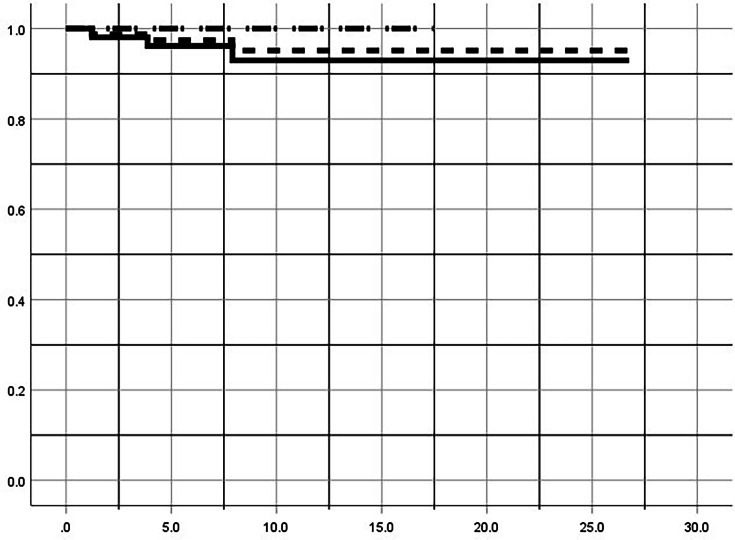
Kaplan-Meier survivorship is shown for all hips (dashed), anteversion (solid), and retroversion (dashed/dotted). Survivorship is shown on the y-axis and time (years) on the x-axis.

Overall survivorship free from conversion to arthroplasty was 97% (95% CI 94 to 100) at five years, and 95% (95% CI 89 to 100) at 10, 15, and 20 years (Figure 2). In excessively anteverted femora, the five-, ten-, 15-, and 20-year survivorship was 96% (95% CI 91 to 100), 93% (95% CI 85 to 100), 93% (95% CI 85 to 100), and 93% (95% CI 85 to 100), respectively. In excessively retroverted femora, the five-, ten-, and 15-year survivorship was 100% at all timepoints. The 20-year survivorship in the retroverted cohort could not be calculated because maximum follow-up did not exceed 20 years. Three hips (two patients), all with excessive anteversion, underwent total hip arthroplasty (THA) at a mean 4.3 years (1.2 to 7.9) following FDO. One patient with Ehlers-Danlos syndrome (EDS) underwent staged, bilateral THA. Another patient with underlying Prader-Willi syndrome underwent THA after failure of a concomitantly performed PAO. Elective hardware removal was performed in 81.8% of hips following radiological union. Two hips (2.6%) underwent revision procedure related to delayed/nonunion of the osteotomy, which included revision osteotomy and exchange nailing. Of these two patients, one had an underlying genetic diagnosis of EDS in which collagen abnormalities may have contributed to poor bony healing. Within the entire cohort, 11.7% (7/60) had underlying genetically proven EDS diagnoses. Three hips (3.9%) underwent subsequent arthroscopy, while one hip (1.3%) underwent subsequent combined hip arthroscopy and PAO. Persistent, clinically significant abductor dysfunction and limp related to trochanteric nail insertion was not observed by the senior author beyond six months postoperatively.

## Discussion

The current study characterizes long-term PROMs and survivorship free of conversion to arthroplasty following FDO for symptomatic excessive femoral anteversion or retroversion at mean ten-year follow-up. The mean mHHS improved by 28.1 points postoperatively (see Table IV). The proportion of MCID, SCB, and PASS achievement was 94% (72 of 77), 84% (65 of 77), and 87% (67 of 77), respectively. Regardless of torsional deformity, the majority (88.7%) reported themselves to be either ‘very much improved’ or ‘much improved.’ Overall, 92.7% of anteverted and 85.7% of retroverted patients are satisfied with their current symptomatic state. Long-term joint preservation was observed independent of version deformity, with 95% survivorship free of conversion to arthroplasty at ten, 15, and 20 years. This included 100% survivorship in femoral retroversion. To the authors’ knowledge, these results reflect the largest cohort with the longest mean follow-up reported to date.

Several retrospective case series demonstrate positive short-term results utilizing intramedullary fixation techniques that were similar to outcomes reported here at long-term follow-up. The mean mHHS improvement of 23.9 was seen in 37 hips (15 increased anteversion, 22 retroversion) at a mean 24-month follow-up.^24^ Overall, 89% of hips achieved the MCID for mHHS utilizing a threshold defined by a cohort undergoing primary hip arthroscopy.^34,35^ In the current study, a mean 28.1 mHHS improvement was seen at mean ten-year follow-up. Previous institutional work demonstrated average mHHS improvement of 29 at a mean of 6.5 years.^22^ Future work is necessary to define the MCID threshold for mHHS in those undergoing FDO. Depending on this threshold, the difference in mHHS magnitude may suggest continued patient pain and functional improvements up to mean 6.5 years postoperatively that is maintained at mean ten years. Among 33 hips with femoral retroversion (mean preoperative version of -3.1°) and mean 19.8-month follow-up, Mastel et al^23^ found 97% of patients reported subjective improvement in pain and satisfaction. Similar overall satisfaction was observed in our retroverted cohort, including 95.2% reporting improvement and 85.7% with acceptable current symptomatic states.

One disadvantage of the described technique is potential damage to the hip abductor tendinous insertion secondary to reamer trauma/nail insertion. Care is taken to establish a trochanteric starting point at the posterosuperior ‘bare area’/’bald spot’ to minimize abductor damage.^36^ Plate osteosynthesis is also not without potential drawbacks, often requiring more invasive approaches with a larger biological insult on the surrounding soft-tissue envelope that may have implications for infection and/or bony union. Additionally, a period of modified weightbearing is typically required. Scarce FDO outcome data exists in non-neuromuscular populations,^37^ making intramedullary and plate fixation technique comparisons difficult. At a mean follow-up ranging from 12 to 48 months, PROMs have been observed to significantly improve after FDO with plate osteosynthesis.^25,38,39^ Rigling et al^39^ examined 25 hips undergoing FDO with plate osteosynthesis combined with hip arthroscopy. No nonunion was observed, and the authors concluded the procedures to be ‘safe and reliable.’ Kamath et al^40^ reported a single revision for delayed union in a chronic smoker using a plating technique. Lerch et al^25^ reported 80% of patients undergoing FDO with plate osteosynthesis would have surgery again. This was similar to the positive PGIC/PASS responses in the current study utilizing an intramedullary fixation technique. Intramedullary nailing for femoral shaft fractures is generally associated with high union and low complication rates.^41,42^ In the current study, two patients had delayed/nonunion necessitating additional surgical intervention.

While most patients improved postoperatively and found their current symptomatic state satisfactory, a small proportion remain unsatisfied. The reasons for this are likely multifactorial. While individual hip structural complexity certainly plays some role, especially if incompletely addressed,^1-3^ our subanalysis demonstrated no difference in mHHS change or proportion of positive PASS/PGIC responses when stratified by concurrent hip preserving surgery. When stratified by specific concurrent preservation procedure, there was no difference in proportion of positive PASS/PGIC responses despite significant variance in mHHS. These differences in mHHS may or may not be clinically meaningful. Using a previously defined MCID of 8 for mHHS in the setting of hip arthroscopy,^34^ the observed variation between FDO in isolation compared with with hip arthroscopy (25.5 vs 26.5, respectively) is likely not clinically relevant. Based on unpublished institutional data, the MCID threshold for mHHS in the setting of PAO is 18. As such, mHHS differences observed between FDO alone and with concurrent PAO (25.5 vs 37.1, respectively) also may not be clinically important. To our knowledge, MCID thresholds for mHHS have not been defined for TDO. Although not captured, activity demands placed on an individual hip undoubtedly contribute to PROM responses. Hip preservation surgeries are performed in patients with diverse clinical characteristics, ranging from the high-level athlete to the middle-aged adult with minimal/mild degenerative joint changes. A growing body of research is highlighting the role of expectations in surgical outcomes, reiterating the importance of setting appropriate preoperative expectations.^43,44^

The current study has limitations. Some degree of recall bias may be present in PROM responses, potentially skewing outcomes as captured by these metrics. Hip ROM assessment was performed by the senior author without routine goniometer use. As this inevitably has some interobserver variability, reported internal and external rotation magnitudes should be interpreted as such. While postoperative CT scans to validate attainment of desired amount of rotational correction would be educational, exposure to unnecessary ionizing radiation could not be justified in patients who were otherwise doing well clinically. As such, these studies were not routinely obtained. Although the senior author’s indication for performing concurrent FDO and PAO remains unchanged over time, the indication for concurrent FDO and hip arthroscopy has changed over the course of his practice. Previously, concurrent arthroscopy was performed in the setting of a labral tear. More recently, however, correction of the underlying structural deformity (in the form of femoral version abnormality) is believed to be sufficient as it offloads the labrum and has a low reoperation rate. Unfortunately, there is no uniform agreement regarding ‘normal’ femoral version. Normal reported values for femoral anteversion are highly variable, including cutoff values for increased and decreased version.^3,8,14,45-47^ While multiple methods with excellent inter- and intraobserver reliability have been described to obtain this measurement, torsion values demonstrate variability depending on the method employed and with increasing anteversion deformity.^30^ Future, multicentre prospective research efforts utilizing quantitative data and outcome measures are needed to better define this heterogeneous patient population, as well as further delineate targets for magnitude of femoral version rotational correction.

In conclusion, awareness of underlying femoral version abnormalities is key in effective hip preservation surgery. The current results support FDO, in isolation or with concurrent hip preservation procedure(s), to correct symptomatic excessive femoral anteversion or retroversion. This technique provides significant improvements in pain and function at mean ten-year follow-up. In appropriately selected patients, the vast majority of patients report postoperative improvement, and find their current symptomatic state satisfactory. The procedure effectively preserves the native hip, with 95% long-term survivorship free of conversion to arthroplasty at 10, 15, and 20 years.

**Take home message**

- Femoral derotational osteotomy (FDO) to correct symptomatic excessive femoral anteversion or retroversion - performed in isolation or with concurrent hip preservation procedure(s) - provides significant improvements in pain and function at a mean ten-year follow-up.

- In appropriately selected patients, the vast majority of patients report postoperative improvement, and find their current symptomatic state satisfactory. FDO effectively preserves the native hip, with 95% long-term survivorship free of conversion to arthroplasty at ten, 15, and 20 years.

## Data Availability

The datasets generated and analyzed in the current study are not publicly available due to data protection regulations. Access to data is limited to the researchers who have obtained permission for data processing. Further inquiries can be made to the corresponding author.
